# Xanthomatous Hypophysitis Mimicking a Pituitary Adenoma: Case Report and Review of the Literature

**DOI:** 10.1155/2010/195323

**Published:** 2010-07-08

**Authors:** Laura Aste, Mattia Bellinzona, Veronica Meleddu, Graziella Farci, Cristina Manieli, Umberto Godano

**Affiliations:** ^1^Department of Neurosurgery, Brotzu Hospital, Via Peretti 1, 09100 Cagliari, Italy; ^2^Department of Pathological Anatomy, Brotzu Hospital, 09100 Cagliari, Italy

## Abstract

*Background*. Hypophysitis is an inflammatory disease of the pituitary gland that may mimic pituitary tumors clinically and radiologically. *Case Description*. We report a case of a xanthomatous hypophysitis initially diagnosed as pituitary adenoma. A 31-year-old woman presented with headache, diabetes insipidus, and amenorrhea. A head CT scan showed no intrasellar changes, while an MRI scan showed a sellar cystic mass. An endocrinological work up revealed mild hypocortisolism and diabetes insipidus (DI). Transsphenoidal surgery was performed. The intraoperative histological examination suggested a pituitary adenoma. The removed tissue showed central necrosis surrounded by accumulation of foamy cells and xanthomatous epithelioid cells. The patient made an uneventful postoperative recovery, Nevertheless, DI persisted and the adenohypophysis hypofunction did not recover. *Conclusion*. We describe an unusual inflammatory lesion of the pituitary gland mimicking an adenoma. A high level of clinical suspicion of inflammatory disorders is necessary for correct diagnosis and optimal management.

## 1. Introduction

Inflammatory disorder of the pituitary gland (hypophysitis) is a rare but increasingly recognized cause of pituitary dysfunction [1] that should be considered in the differential diagnosis of sella turcica lesions [1]. Secondary hypophysitis is rare, while primary hypophysitis has traditionally been classified as lymphocytic (LH), granulomatous (GH), and xanthomatous hypophysitis (XH) [1–3]. 

Xanthomatous hypophysitis (XH) has been recently described, and it is the rarest of the three types [1]. XH is characterized by mixed inflammatory cell infiltrates consisting of foamy histiocytes, plasma cells, and small round mature lymphocytes infiltrating the anterior pituitary gland [3–6].

Clinically the lesion may mimic a pituitary neoplasm as a result of mass effect and hypothalamic-hypophyseal endocrine dysfunction [6]. We report here the case of a young woman who presented with a sellar mass on a head MRI and endocrine dysfunction.

## 2. Case Report

A 31-year-old women was admitted to our department with a one-month history of headache and diabetes insipidus (DI). At admission, the patient was found to suffer from amenorrhea. Furthermore, the patient had a positive history for ulcerative colitis under medical treatment.

At the first physical examination, the patient appeared in good general conditions, had no fever, no hypertension, no signs of meningism, and no neurological deficits. The first pituitary function work up showed gonadotropic hormons in the lower normal range (NR): cortisol levels: at 08 a.m. = 12.1 ug/dl (NR = 8.0–25.0.), at 02 p.m. = 3.6 ug/dl (NR = 1.0–17), at 10 p.m. = 1.6 ug/dl (NR = 1.0–5.0); PRL = 13.8 ng/mL (NR = 3.0–27.0); FSH = 1.9 mUI/mL (NR = 2.0–13.0 follicular phase); LH = 2.0 mUI/mL (NR = 2.0–12.0 follicular phase); progesterone = 0.27 ng/mL (NR = 0.10–1.00 follicular phase); estradiol = 26.4 pg/mL (NR = 20.0–200.0 follicular phase); TSH = 0.24 uIU/mL (NR = 0.25–5.00); FT3 = 2.49 pg/mL (NR = 2–4); FT4 = 1.25 ng/dl (NR = 0.6–1.7); GH = 1.1 ng/mL (NR = 0–4.7).

A second preoperative endocrinological work up one month later showed reduced corticol levels, that is, cortisol (08:00 a.m.) = 0.9 ug/dl; FSH = 7.9 mUI/mL; PRL = 10.3 ng/mL; LH = 10.1 mUI/mL; progesterone = 0.19 ng/mL; estradiol = 7.9 pg/mL; FT3 = 2.27 pg/mL; FT4 = 1.41 ng/dl; GH = 0.8 ng/mL. Inflammatory indices such as WBC and haptoglobin were slightly increased (11.45e3/uL and 282 mg/dl, resp.). Plasma osmolarity was normal. Urine tests, including electrolytes, were within normal limits, except for low levels of phosphate, BUN, creatinine, and magnesium.

**Figure 1:**
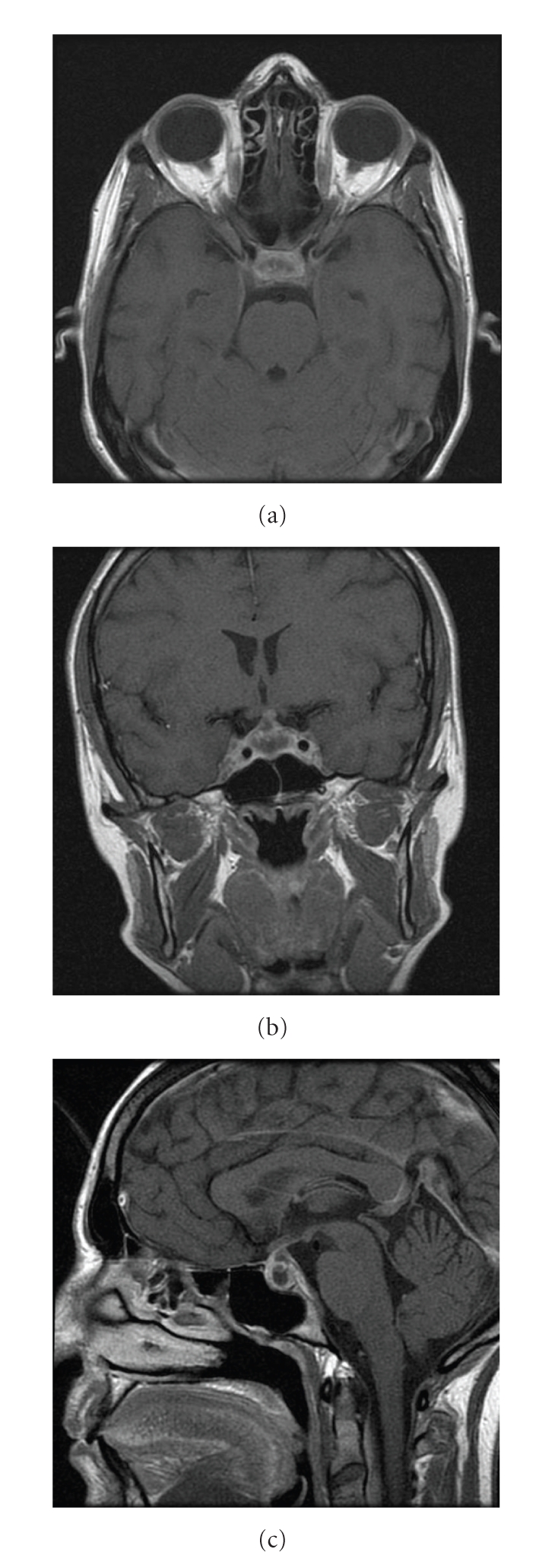
Pre-operative axial (a), coronal (b) and sagittal (c) Gadolinium-enhanced T1-w MR images showing the cystic lesion in the sella.

A contrast head CT scan was described as normal. A first head Gadolinium MRI showed an increased volume of the pituitary gland with mild compression of the optic chiasm. A second head MRI (multiplanar FSE −/+ Gad, GRE-T2*) with thin slices on sellar region revealed a widening of the sella occupied by a solid lesion with necrotic core and a hemorrhagic spot. The lesion was measured to be 21 × 20 × 12 mm. It extended into the suprasellar cistern determining a minimal displacement of the optic chiasm (Figure 1).

Indication for transsphenoidal surgery was given. Prior to surgery the patient underwent a complete pituitary function work-up (see above). An ophtalmological work-up revealed no visual fields abnormalities. A preoperative thyroid gland echography showed increased volume with small nonechogenic nodules. An abdomen CT scan showed intestinal parietal thickening and pseudopolypoid nodular disease compatible with ulcerative images.

Endovenous intranasal Desmopressin (DDAVP-Minirin) was started few days after the admission, and prophylactic Hydrocortisone was given 48 hours prior to surgery.

**Figure 2:**
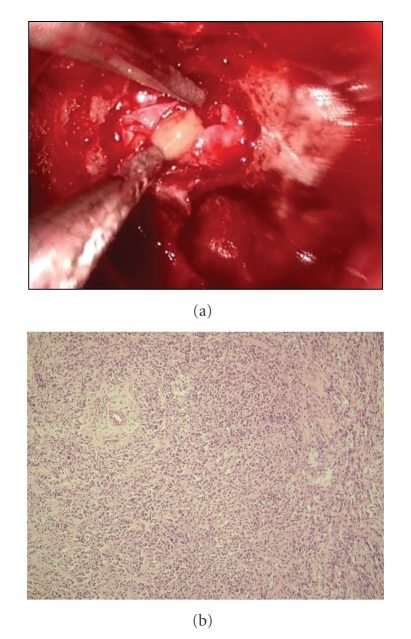
(a) Intraoperative image showing the pus-like fluid drained at dural opening. (b) Intraoperative frozen section showing round and polygonal cells, with a diffuse growth pattern; no evidence of inflammatory cells or foamy histiocytes.

Surgical exploration via the endoscopic transsphenoidal route revealed a cystic lesion containing pus-like fluid, which was drained after dural opening (Figure 2(a)). Tissue samples were taken for histological examination from the cyst wall. The intraoperative histopathological examination indicated a pituitary adenoma (Figure 2(b)). 

**Figure 3:**
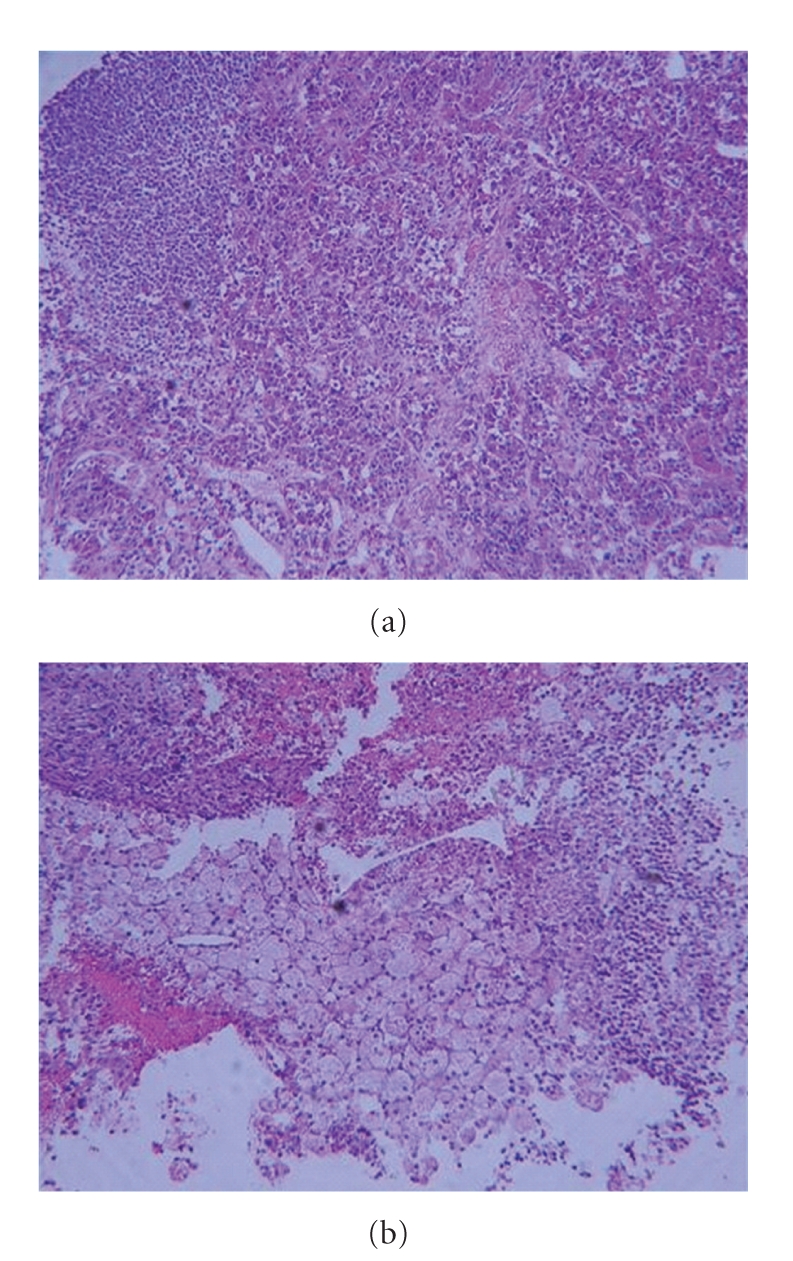
Hematoxylin-Eosin stain. Fragments of intact, normal pituitary gland infiltrated by foamy histiocytes.

**Figure 4:**
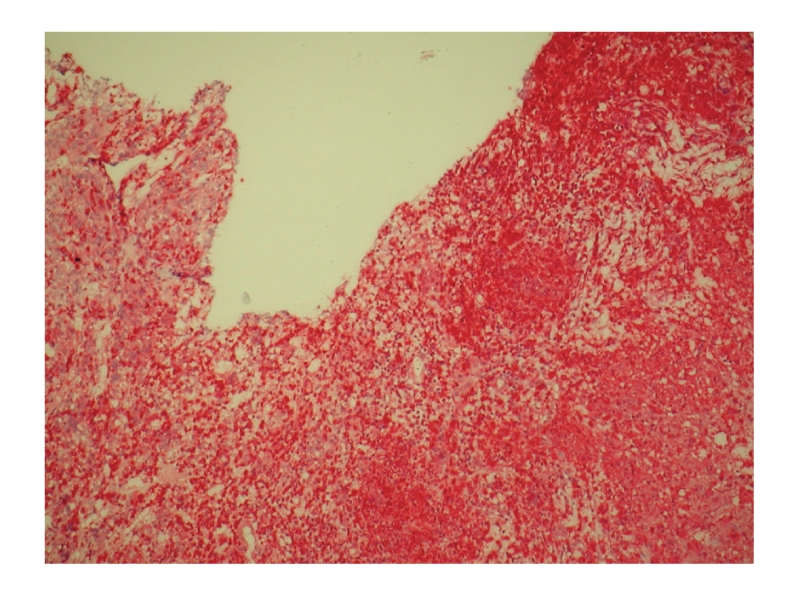
Postoperative immunohistochemical stain. Chronic inflammatory cells reaction with collections of foamy histiocytes (IHC: CD68, fast red).

The postoperative full histopathological work-up revealed an inflammatory infiltrate of numerous foamy histiocytes, multinucleated giant cells containing cholesterol clefts, and lymphocytes infiltrating between residual nests of normal pituicytes (Figure 3). On immunohistochemical sections, the infiltrating foamy cells, were strongly positive for CD68, a macrophage marker (Figure 4). There was no evidence of associated adenomas, granulomas, Langherans' cells or neutrophilic exudates. No haemosiderin was seen. The rupture of a Rathke's cleft cyst was excluded by the pathologist as no cylindric ciliated epithelium could be seen.

There were no surgical complications. The patient suffered from transient rhinoliquorrea. Pituitary function did not improve; 5 days after surgery she underwent a complete endocrinological work-up with the following findings: cortisol at 08 a.m. = 14.5 ug/dl, at 12 a.m. = 11.9, and at 12 p.m. = 12.4; PRL = 1.6 ng/dl; FSH = 6.1 mUI/mL; LH = 3.0 mUI/mL; TSH = 0.21uUI/ml, FT3 = 1.69 pg/mL, FT4 = 1.5 ng/dl; GH = 0.3 ng/mL, ACTH = 5.9 pg/mL (NR = 5–77); IGF1 = 53 ng/mL (NR = 115–500); C-peptide = 2.2 ug/mL (NR = 0.8–3.0). The patient continued to take DDAVP, and she was started on cortone acetate 25 mg 1/2 cp × 3/die. Postoperative electrolytes were always within a normal range. Urine output required one daily administration of vasopressin (nasal spray). Hormonal substitution therapy after discharge was prescribed. The headache resolved after surgery.

**Figure 5:**
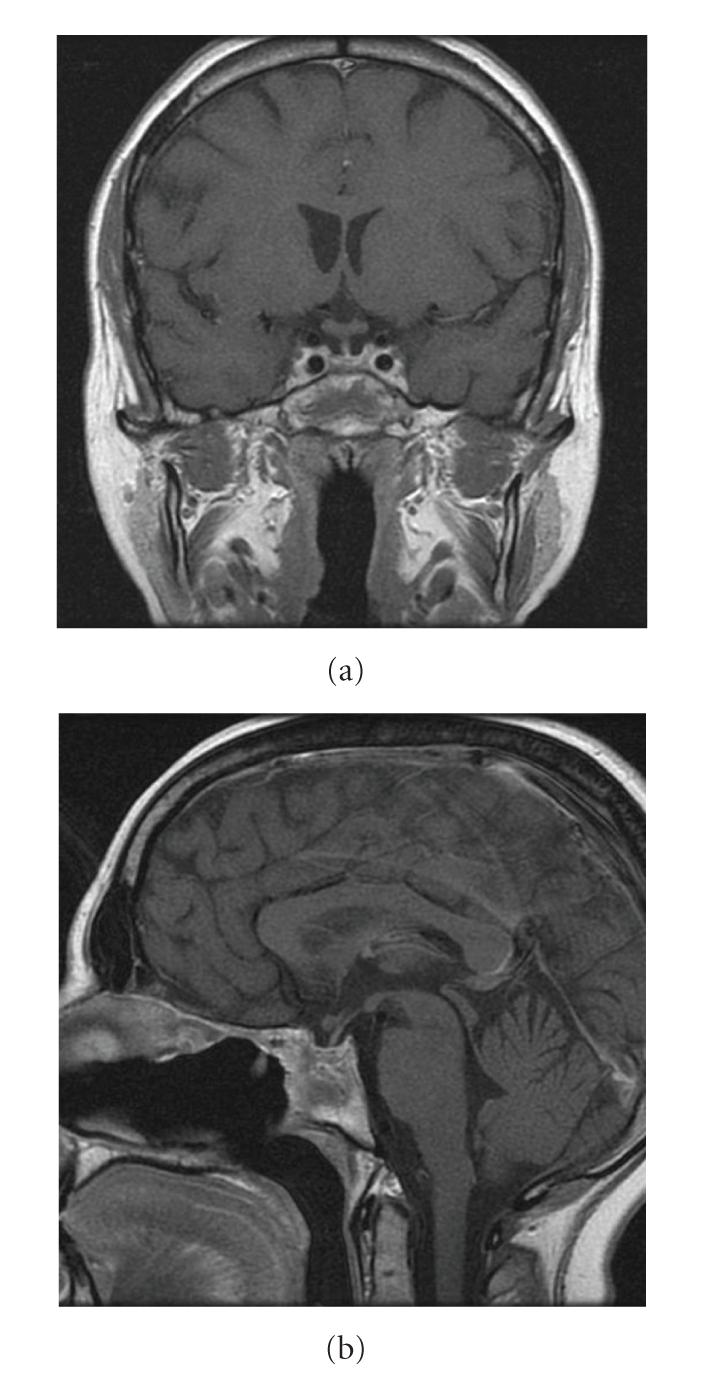
Postoperative coronal (a) and sagittal (b) T1-w MR images, showing no pathological changes in the sellar region.

The patient underwent an endocrinological work-up at 1, 3, and 8 months after surgery; serum prolactin gradually increased from 5.25 to 8.9 ng/mL. Cortisol level increased from 0.9 ug/dl preoperativly to 18 ug/dl at 3 months and 31 ug/dl at 8 months postoperatively. Urinary cortisol levels also increased. FSH, LH, and TSH remained in the lower normal range while FT3 and FT4 slightly increased. GH = 0.4 ng/mL at one month postoperatively. PCR was 3.19 at the follow-up. Elecrolytes were within normal range. A postoperative Gadolinium-enhanced head MRI at 8 months showed a normal pituitary gland with no pathological changes (Figure 5). 

Eight months postoperatively, the patient still required glucocorticoid and estrogen replacement therapy as amenhorrea still persisted. Cortone acetate has been progressively reduced 12 months after surgery with cortisol levels remaining within normal range. Recently, the patient decided to stop estroprogestinic drugs because of a pregnancy desire; amenhorrea was still present. She must also continue DDAVP.

## 3. Discussion

Hypophystis is an infrequent inflammatory disease of the pituitary gland that may be misdiagnosed as a pituitary adenoma or other sellar masses as a Ratcke's cleft cyst. Histologically hypophysitis includes three distinct histopathological subtypes: Lymphocytic Hypophysitis (LH), Granulomatous Hypophysitis (GH), and Xanthomatous Hypophysitis (XH) [1–3]. Some authors classify the xanthogranulomatous (XGH) and necrotizing hypophysistis (NH) as autonomous entities [2, 3]. XGH has been recently defined as a new category by Tashiro et al. for the presence of multinucleated giant cells [3], and it has also been described in association with adamantinomatous craniopharyngiomas [4]. In terms of the site of inflammation, hypophysitis is also subdivided into adenohypophysitis and infundibuloneurohypophysitis [3]. The latter is thought to be a different entity from LH because inflammation is confined to the hypothalamic neurohypophyseal system causing DI [3, 7, 8]. For this reason, it is thought to be one of the causes of idiopathic DI [7, 8]. On MR imaging, a thickening of the neurohypophysis or pituitary stalk is usually seen [7]. Since in idiopathic DI a lack of normal early enhancement of the posterior pituitary is often described, the etiopathogenetic hypothesis is focused on the decreased posterior pituitary vascularity due to congenital lack, to poor development of the posterior pituitary arterial supply, or to secondary changes of vascular destruction [8]. 

LH is the most common type and is characterized by infiltration of the anterior pituitary gland with lymphocytes and plasma cells and by fibrosis, most commonly observed in females during or after pregnancy [7, 9]. GH does not have a high predominance in females and is characterized by granulomas with epithelioid histiocytes and multinucleated giant cells; it is different from systemic giant-cell disorders such as tuberculosis, sarcoidosis, or syphilis [7]. Few cases of GH with giant cell reaction caused by the rupture of a Rathke's cleft cyst have been described [10]. NH consists of inflammation with necrosis of the hypothalamus, pituitary stalk, adenohypophysis, and infundibuloneurohypophysis, often accompanied by hypofunction and DI [3]. 

Xanthomatous hypophysitis (XH) is a rare inflammatory disease that usually involves the anterior lobe of the pituitary gland [1]. Since the first report in 1998 by Folkerth et al. [11], a few cases of XH have been reported in literature [3–6, 12]. Clinical and laboratory findings suggest an autoimmune basis for primary hypophysitis [2, 11]. The patient presented in this report had a history of ulcerative colitis, supporting this hypothesis, although autoimmune disorders are predominantly associated with lymphocytic hypophysitis [3]. 

XH has been shown to have a predilection for young women [4]. Headache is a common complaint at presentation, while visual disturbances are uncommon [1, 2]. 

The clinical symptoms of primary hypophysitis are mainly due to inflammatory irritation of sellar and parasellar structures. 

DI, probably caused by inflammatory infiltration rather than by compression of the posterior lobe and pituitary stalk, is rare in XH because inflammation is predominant in the anterior pituitary lobe while it occurs in 50% of patients suffering from LH and GH and corresponds to pituitary stalk thickening in MRI studies [1, 7]. It can be assumed that in XH a circumscribed anterior pituitary lesion may lead to compression of the pituitary gland without alteration of pituitary stalk and optic chiasm [1]. Moreover, DI is not often seen at the clinical onset of pituitary adenomas (PA). MR imaging is the procedure of choice in the evaluation of sellar masses, although diagnosis by MRI alone can only be suspected: most XH cases appear as an enlargement of the pituitary gland with hypointense and round cystic lesions in T1-weighted images [1, 2, 4, 5]. At surgery, thick fluid material is found within the cystic lesion [6, 12]. 

Gutenberg et al. (ANJR, 2009) proposed a radiologic score to distinguish autoimmune hypophysitis from non secreting PA [13]. In the differential diagnosis between hypophysitis and PA, the peculiar radiologic features of the first ones are a symmetric enlargement of pituitary gland, a homogeneous appearance both on pre- and post-Gadolinium images, and an intense contrast enhancement. In contrast, PA are typically asymmetric showing heterogeneous enhancement and had a lower Gadolinium uptake. Moreover, a thickened pituitary stalk is typical for autoimmune hypophysitis [7, 13, 14]. Others pathological entities to be considered in the differential diagnosis are pituitary abscesses (nowadays uncommon), secondary hypophysitis and “noninfectious” granulomatous processes.

XH is characterized histologically by an inflammatory infiltrate of foamy histiocytes [3, 4, 6]. The basic structure of anterior pituitary is generally preserved [2, 6], without alteration of the pituitary stalk and optic chiasm [2]. Several biopsies should be performed for an exact histological diagnosis.

The overall prognosis for XH is good, but improvement of pituitary function after transsphenoidal surgery has been reported in less then 50% of the cases in the literature [1, 3]. Chronic inflammation may result in destruction and fibrosis of the pituitary gland [4].

In their initial description of XH, Folkhert et al. [11] reported two 30-year-old women presenting with menstrual dysfunction and a 12-year-old girl presenting with nausea, headache, and symptoms of DI. Subsequently, Deodhare et al. reported about a 43-year-old female patient presenting with hyperprolactinemia, GH and cortisol deficiency, and a DI. Cheung et al. and Tashiro et al. reported about three more cases of female patients affected by XH diagnosed after a transsphenoidal surgery. 

Burt et al., while reporting two cases of XH in two young men, have described the first documented case of XH with full recovery of pituitary function after surgery [4].

Gutenberg et al. [1] in a study involving 21 lymphocytic, 6 granulomatous, and 4 XH cases have shown that endocrinological and neuroradiological improvement under pre- or post-operative high-dose corticosteroids is most often incomplete and transient. A prospective trial of glucocorticoid use in LH showed that methylprednisolone reduced MRI mass in seven out of nine patients and improved anterior pituitary function in four patients. As far as XH is concerned, they showed no benefit after either pre- or post-surgical corticosteroids, while 50% of XH cases had an improvement of anterior pituitary dysfunction shortly after surgery and none suffered from endocrinological deterioration. Our paper confirms the tendency of XH hypophysitis to cause a long lasting-though mild-hypopituitarism.

While steroid therapy seems to be less effective in XH compared to other kinds of primary hypophysitis, surgical exploration is recommended in almost all pseudotumoral hypophysitis in order to relieve symptoms and secure a diagnosis [1, 14]. Headache seems to improve in most of patients who underwent surgery [1].

A long-term follow-up on XH patients is not yet available. This will be useful to compare the effectiveness of different therapeutic strategies. We suggest that in case of radiological or serological suspicion of inflammatory disease of the pituitary gland, a confirmatory biopsy through a transsphenoidal approach should be performed. In case of compression of sellar and suprasellar structures, surgical removal should be recommended [12]. 

## Abbreviations

XH: Xanthomatous Hypophysitis
LH: Lymphocytic Hypophysitis
GH: Granulomatous Hypophysitis
CT: Computed Tomography
MRI: Magnetic Resonance Imaging
DI: Diabetes Insipidus
PA: Pituitary Adenoma.
